# Validity of SyMRI for Assessment of the Neonatal Brain

**DOI:** 10.1007/s00062-020-00894-2

**Published:** 2020-03-11

**Authors:** Victor Schmidbauer, Gudrun Geisl, Mariana Cardoso Diogo, Suren Jengojan, Vsevolod Perepelov, Michael Weber, Katharina Goeral, Florian Lindenlaub, Katrin Klebermass-Schrehof, Angelika Berger, Daniela Prayer, Gregor Kasprian

**Affiliations:** 1 Department of Biomedical Imaging and Image-guided Therapy, Medical University of Vienna, Währinger Gürtel 18–20, 1090 Vienna, Austria; 2 Department of Pediatrics and Adolescent Medicine, Comprehensive Center for Pediatrics, Medical University of Vienna, Währinger Gürtel 18–20, 1090 Vienna, Austria

**Keywords:** Magnetic resonance imaging, Newborn, Software, Sensitivity, Specificity

## Abstract

**Purpose:**

The purpose of this study was to assess the diagnostic accuracy of T1-weighted and T2-weighted contrasts generated by the MR data postprocessing software SyMRI (Synthetic MR AB, Linköping, Sweden) for neonatal brain imaging.

**Methods:**

In this study 36 cases of neonatal MRI were retrospectively collected, which included T1-weighted and T2-weighted sequences as well as multi-dynamic multi-echo (MDME) sequences. Of the 36 neonates 32 were included in this study and 4 neuroradiologists independently assessed neonatal brain examinations on the basis of conventional and SyMRI-generated T1-weighted and T2-weighted contrasts, in order to determine the presence or absence of lesions. The sensitivity and specificity of both methods were calculated and compared.

**Results:**

Compared to conventionally acquired T1 and T2-weighted images, SyMRI-generated contrasts showed a lower sensitivity but a higher specificity (SyMRI sensitivity 0.88, confidence interval (CI): 0.72–0.95; specificity 1, CI: 0.89–1/conventional MRI: sensitivity: 0.94, CI: 0.80–0.98; specificity: 0.94, CI: 0.80–0.98).

**Conclusion:**

The T1-weighted and T2-weighted images generated by SyMRI showed a diagnostic accuracy comparable to that of conventionally acquired contrasts. In addition to semiquantitative imaging data, SyMRI provides diagnostic images and leads to a more efficient use of available imaging time in neonatal brain MRI.

## Introduction

Brain imaging is essential in neonatal neuropediatric diagnostics [1]. In neonates, magnetic resonance imaging (MRI) serves as a reliable modality for the detection of brain pathologies, as well as for the assessment of myelination [2–6]. Based on conventional MRI findings, substantial information about the future course of neurodevelopment can be obtained [2, 7–9]. Apart from deficient myelination, brain injury, e.g., due to hemorrhage, asphyxia, or infarction during the prenatal and neonatal period, may severely impair brain maturation [6, 8, 10–12]. Therefore, a precise visualization of pathological changes must be the aim of imaging methods, in order to ensure an optimal individual diagnosis and follow-up.

Conventional MRI is a meaningful, yet highly time-consuming method, for brain imaging. Primarily in a pediatric cohort, a long examination time may overtax patient compliance. Hence, it is of paramount importance to develop imaging techniques that enable a reduction of acquisition time.

Based on a single, quantitative, multi-dynamic multi-echo (MDME) sequence, the MR data post-processing software SyMRI (Synthetic MR AB, Linköping, Sweden) enables the generation of various MR contrasts, such as T1-weighted, T2-weighted, proton density (PD)-weighted, and inversion recovery [13], which bears the potential to reduce examination time [14–16]. Moreover, SyMRI allows the generation of quantitative maps, which facilitate the assessment of myelination and brain development [4, 14, 17]. Using the MDME sequence, tissue-specific parameters, such as T1-relaxation constants, T2-relaxation constants, and PD of the brain, can be acquired in less than 6 min [13, 16, 18–24]. Contrary to conventional MRI techniques, repetition time (TR), echo time (TE), and inversion time are not predefined in this method, as these factors are definable and adjustable in retrospect [15, 20]. Subsequently, SyMRI generates the desired contrasts within a few seconds [13]. Thus, it would be conceivable to apply SyMRI in the clinical routine. Previously published data show that T1 and T2-weighted contrasts generated by SyMRI are comparable to those of conventionally acquired MR image data [15, 19]. This technique has already been investigated as a diagnostic tool in a variety of conditions, primarily in neurological diseases [25–28]; however, the majority of studies referred exclusively to adults. Thus, further data on the applicability of SyMRI in children and neonates are of particular importance.

The aim of this study was to evaluate the clinical practicability of SyMRI-generated MR contrasts for the assessment of the neonatal brain. For this purpose, four neuroradiologists, independently rating, assessed a variety of cases based on T1 and T2-weighted images generated by SyMRI and conventional T1 and T2-weighted sequences. The sensitivity and specificity of conventional MRI and the MR data post-processing software SyMRI were calculated and compared in a neonatal cohort.

## Material and Methods

### Ethical Approval

The protocol of this study was approved by the local Ethics Commission and performed in accordance with the Declaration of Helsinki.

### Study Cohort

We retrospectively collected 36 cases of neonatal MRI, which included T1 and T2-weighted sequences, as well as MDME sequences. The data acquisition was performed at the neuroradiology department of a tertiary care hospital between June 2017 and August 2019. All newborns were referred for MRI examination by the Department of Pediatrics and Adolescent Medicine, Division of Neonatology, Pediatric Intensive Care and Neuropediatrics. At this institution, every preterm infant is subjected to MRI at approximately term-equivalent age, regardless of clinical presentation or previous ultrasound findings. Term-born infants were examined postnatally between 2 days and 12 weeks postpartum. Indications for the MRI included, e.g., a diagnosis or suspicion of intraventricular hemorrhage, hypoxic ischemic encephalopathy, and cerebral infarction. By means of the electronic patient documentation system, the clinical information of the subjects was retrospectively obtained. After careful review, a total of 32 neonates, 50% of which were inconspicuous cases (16/32), were included in this study. Of 36 subjects, 4 subjects had to be excluded due to lack of clinical information and 2 of 16 inconspicuous subjects were term-born. Clinical follow-up examinations confirmed an unimpaired neurological development. Clinical characteristics and demographic information of the included neonates are shown in Table 1.

**Table 1:** Demographics and clinical characteristics

	Neonates *n* = 32
Characteristics/demography	
Male/female	16/16
Term born/preterm	8/24
Gestational age (weeks)^a^	27 + 5 (23 + 3–41 + 5)
*Clinical diagnosis*	
Inconspicuous^b^	*n* = 16
Hemorrhage^b,c^	*n* = 9
Expired infarction^b^	*n* = 2
HIE^b^	*n* = 3
PVL^b^	*n* = 1
Venous vessel malformation^b^	*n* = 1

*HIE* hypoxic-ischemic encephalopathy, *PVL* periventricular leukomalacia

^a^Data represented as median and range

^b^Data represented as total number

^c^Including hyperacute, acute, subacute, and chronic intraventricular, cortical, subcortical, parenchymal, and subdural hemorrhage

### Data Acquisition, MDME Sequence, and MR Post-processing

In order to prevent movement artifacts, infants were fed 30 min prior to the MRI examination and bedded in a vacuum mattress. In cases of routine MRI due to prematurity, no sedation was used. In cases of suspected or diagnosed brain pathologies, a sedation using chloral hydrate (30–50 mg/kg) or chloral hydrate combined with midazolam (0.1 mg/kg) was used. All included subjects were examined using a standardized neonatal MRI protocol (T1 spin echo [SE] sequence [single plane], T2 turbo spin echo [TSE] sequence [three orthogonal planes], diffusion-weighted imaging [DWI] sequence, susceptibility-weighted imaging [SWI] sequence, T1 3D sequence) on a Philips Ingenia (Philips Healthcare, Best, The Netherlands) 1.5 T MR system. In addition, an MDME sequence (single plane) was acquired. The sequence determines T1 and T2-relaxation constants, as well as PD of the examined tissue, by applying two repeated acquisition phases [13, 29]. During the first phase, a slice-selective saturation pulse (flip angle: 120°) was used to saturate one slice. During the second phase, slice-selective excitation pulses (flip angle: 90°) and slice-selective refocusing pulses (flip angle: 180°) were used to generate a train of spin echoes for another slice [13, 16, 29]. Based on the mismatch between the saturated slice and the image slice, a matrix with various effects of T1- and T2-relaxation rates was acquired [13, 29]. Echo trains characterized by different saturation delays allowed the T1- and T2-relaxation parameters to be estimated [13, 16, 29]. The ascertained T1-relaxation constants also enabled the calculation of the local radiofrequency field (B1), which allowed the correction of flip angle deviations. Based on the acquired relaxation parameters and B1, the PD can be calculated [16]. The MDME sequence-based MR post-processing was performed by applying SyMRI (Version 11.1.5) to generate T1-weighted (preset TR = 650 ms; preset TE = 10 ms) and T2-weighted (preset TR = 4500 ms; preset TE = 100 ms) contrasts. Table 2 gives an overview about the technical features of the individual sequences.

**Table 2:** MRI sequences and technical features

Sequence	Plane	Matrix (slices)	FOV (mm)	Voxel size (mm)	TR (ms)	TE (ms)	AT
T1 SE	Axial	144 × 115 × 30	120 × 120 × 90	0.83 × 1.05 × 3	400	15	3:07
T2 TSE	Axial	128 × 113 × 34	120 × 120 × 102	0.94 × 1.06 × 3	3000	140	1:48
T2 TSE	Coronal	116 × 103 × 36	110 × 110 × 108	0.94 × 1.06 × 3	3000	140	1:48
T2 TSE	Sagittal	128 × 113 × 36	120 × 120 × 108	0.94 × 1.06 × 3	3000	140	1:48
DWI	Axial	176 × 170 × 28	200 × 200 × 92	1.14 × 1.15 × 3	4066	90	1:34
SWI	Axial	200 × 138 × 90	170 × 139 × 90	0.85 × 1 × 2	51	12	3:35
T1 (3D)	Sagittal	160 × 160 × 99	120 × 120 × 99	0.75 × 0.75 × 2	25	7.6	3:46
MDME	Axial	224 × 159 × 22	200 × 165 × 109	0.9 × 1 × 4	3309	13	5:24

*AT* acquisition time, *DWI* diffusion-weighted imaging, *FOV* field-of-view, *MDME* multi-dynamic multi-echo sequence, *SE* spin echo, *SWI* susceptibility-weighted imaging, *TE* echo time, *TR* repetition time, *TSE* turbo spin echo

### Assessment of the Neonatal Brain

**Fig. 1:**
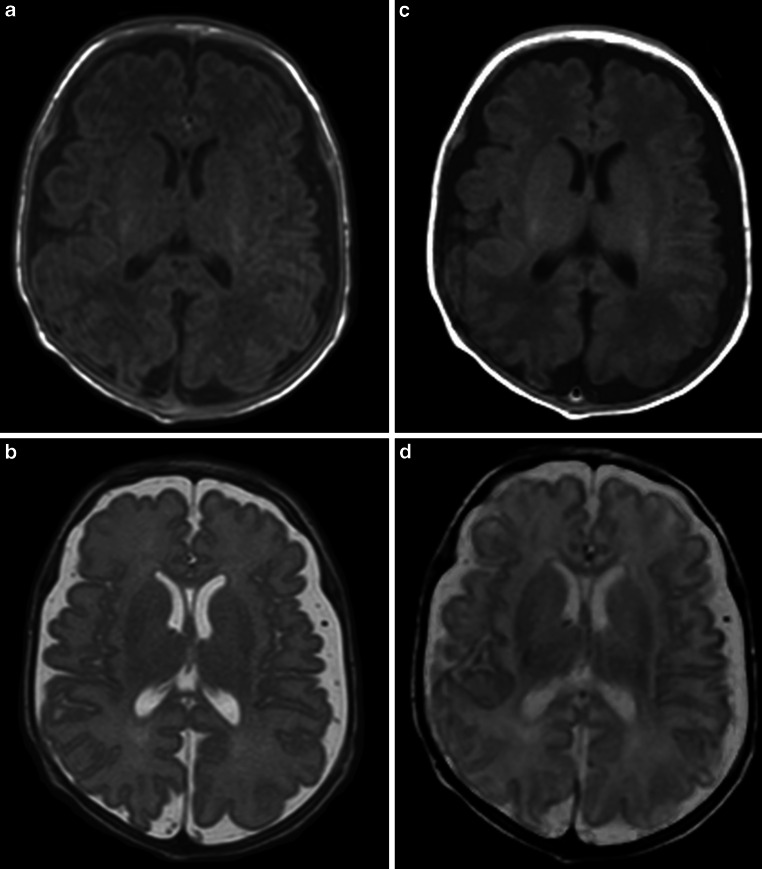
Inconspicuous case: brain of a former preterm infant (24 + 4 weeks). The left column (**a**, **b**) shows conventionally acquired T1-weighted (**a**) and T2-weighted contrasts (**b**). The right column (**c**, **d**) shows T1-weighted (**c**) and T2-weighted (**d**) contrasts generated by the MR data post-processing software SyMRI

**Fig. 2:**
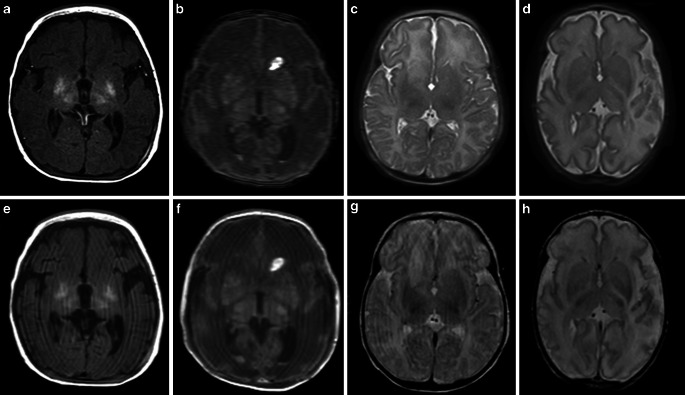
Selection of pathological cases. The *upper row* shows conventional MR contrasts (**a**–**d**). The *bottom row* shows SyMRI-generated MR contrasts (**e**–**h**). **a**, **e** Hypoxic ischemic encephalopathy in a term-born infant (39 + 4 weeks) (T1-weighted contrast). **b**, **f** Former preterm neonate (34 + 6 weeks) with multifocal intracranial hemorrhage (T1-weighted contrast). **c**, **g** Term-born infant (41 + 5 weeks) with expired infarction in the right caudate nucleus (T2-weighted contrast). **d**, **h** Former preterm neonate (32 + 4 weeks) with infarction of the left hemisphere (T2-weighted contrast)

**Fig. 3:**
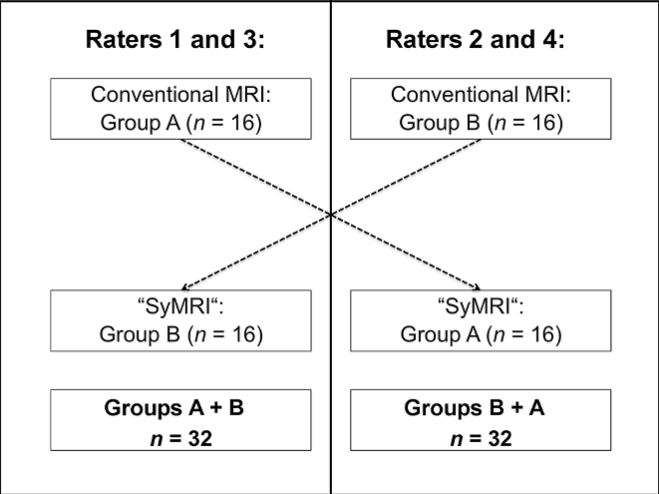
Allocation of the groups to the corresponding raters

In order to compare conventional MR contrasts and SyMRI-generated MR contrasts (Figs. 1 and 2), the included subjects were divided into two groups. Each group (*n* = 16) included 50% inconspicuous cases (8/16) and 50% pathological cases (8/16). Table 3 shows the allocation of pathological cases to the groups. For the assessment of image data, four independent investigators were consulted (rater 1: 15 years of experience in assessing neonatal MRI; rater 2: 6 years of experience in assessing neonatal MRI; raters 3 and 4: limited experience in assessing neonatal MRI). All raters were blinded to the diagnosis of the included neonates. Each group was evaluated by an experienced and a less experienced investigator on SyMRI-generated, as well as on conventionally acquired axial T1- and T2-weighted contrasts. Hence, raters 1 and 3 assessed the first group based on conventional T1- and T2-weighted images, whereas raters 2 and 4 assessed the same group based on SyMRI-generated T1- and T2-weighted contrasts. Conversely, raters 1 and 3 assessed the second group based on SyMRI-generated T1 and T2-weighted contrasts and raters 2 and 4 assessed the same group based on conventionally acquired T1- and T2-weighted images (Fig. 3). Based on the provided image data, raters had to make the correct diagnosis. In addition, the assessing neuroradiologists obtained information about the gestational age at birth of the subjects, as well as the corresponding indication for the MRI examination. The raters were asked about signs of hemorrhage, infarction, or hypoxic-ischemic encephalopathy, if the correct diagnosis was deducible based on the referral. Available follow-up MRI examinations and other imaging modalities allowed the clarification of the correct diagnosis in advance. During the assessment of conventional images, the evaluators had the opportunity to adjust the windowing at their discretion. In the case of SyMRI-generated contrasts, the raters had the option to modulate TR and TE, in order to generate appropriate images.

**Table 3:** Group allocation

Group A^a^		Group B^b^	
Condition/pathology	*n* = 16	Condition/pathology	*n* = 16
Inconspicuous^c^	*n* = 8	Inconspicuous^c^	*n* = 8
Hemorrhage^c^	*n* = 5	Hemorrhage^c^	*n* = 4
Expired infarction^c^	*n* = 1	Expired infarction^c^	*n* = 1
HIE^c^	*n* = 1	HIE^c^	*n* = 2
Venous vessel malformation^c^	*n* = 1	PVL^c^	*n* = 1

*HIE* hypoxic ischemic encephalopathy, *PVL* periventricular leukomalacia

^a^Assessed by raters 1 and 3 on the basis of conventionally acquired T1- and T2-weighted contrasts. Assessed by raters 2 and 4 based on SyMRI-generated T1- and T2-weighted contrasts

^b^Assessed by raters 1 and 3 on the basis of SyMRI-generated T1- and T2-weighted contrasts. Assessed by raters 2 and 4 based on conventionally acquired T1- and T2-weighted contrasts

^c^Data represented as total number

### Statistical Analyses

Statistical analyses were performed using XLSTAT 2017 Version 20.5 (Addinsoft, Paris, France) and SPSS Statistics for Macintosh Version 25.0 (IBM Corporation, Armonk, NY, USA) at a significance level of alpha (*α*) = 5% (*p* < 0.05). Graphs were created using XLSTAT 2017 Version 20.5.

The sensitivity and specificity for both conventionally acquired and SyMRI-generated T1 and T2-weighted contrasts were calculated separately based on the evaluation of experienced and less experienced raters. Furthermore, total values for sensitivity and specificity were calculated on the basis of the assessment of all raters.

An interrater reliability was calculated using the Cohen coefficient (Cκ) and the Fleiss coefficient (Fκ). The Cκ was used to detect concordances between the assessment of raters 1 and 2 (experienced) and raters 3 and 4 (less experienced). The Fκ was used to detect the concordances of the assessment of all raters. According to Landis and Koch, κ was interpreted as follows: κ ≤ 0: poor agreement; 0 < κ ≤ 0.2: slight agreement; 0.2 < κ ≤ 0.4: fair agreement; 0.4 < κ ≤ 0.6: moderate agreement; 0.6 < κ ≤ 0.8: substantial agreement; 0.8 < κ ≤ 1: (almost) perfect agreement [30].

The determined values were complemented by the corresponding 95% confidence interval (CI).

## Results

### Diagnostic Accuracy, Sensitivity, and Specificity

**Fig. 4:**
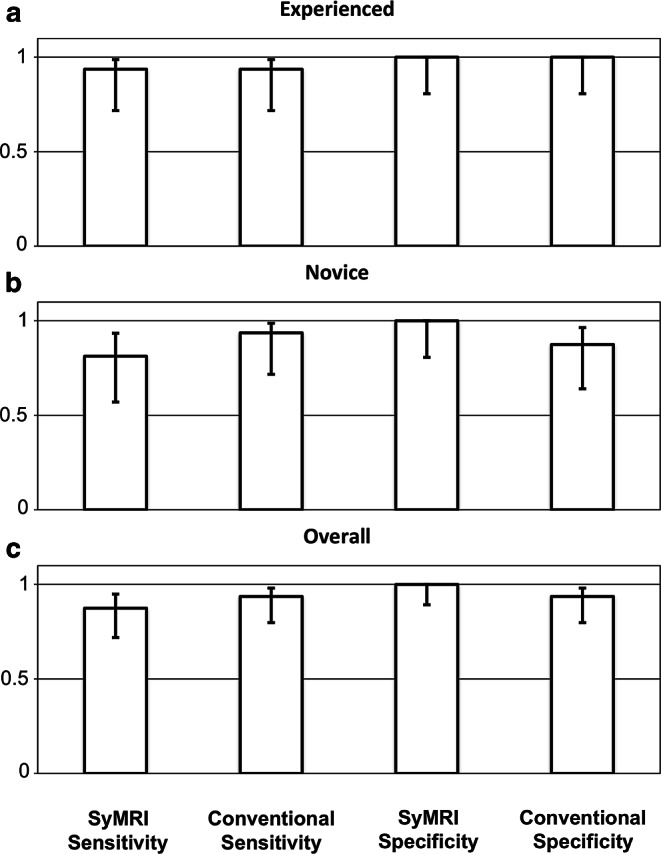
**a** Sensitivity and specificity of conventional MRI and SyMRI on the basis of the assessment of experienced raters. **b** Sensitivity and specificity of conventional MRI and SyMRI on the basis of the assessment of novice raters. **c** Sensitivity and specificity of conventional MRI and SyMRI on the basis of the assessment of all raters

On both conventional and SyMRI-generated T1 and T2-weighted images, 31/32 cases (96.9%) were correctly diagnosed or identified as inconspicuous by raters 1 and 2 (Table 4). With respect to the assessment of experienced raters, the sensitivity and specificity of conventional and SyMRI-generated MR contrasts did not show any differences (SyMRI: sensitivity: 0.94 [CI: 0.72–0.99]; specificity: 1 [CI 0.81–1]/conventional MRI: sensitivity: 0.94 [CI: 0.72–0.99]; specificity: 1 [CI: 0.81–1]; Fig. 4a).

**Table 4:** Incorrectly identified cases

Missed	Rater	Modality	Suspected	Group
Inconspicuous	3^a^	Conventional MRI	HIE	A
Expired infarction	4^a^	SyMRI	Inconspicuous	A
PVL	3^a^	SyMRI	Inconspicuous	B
Inconspicuous	4^a^	Conventional MRI	HIE	B
Expired infarction	1^b^	SyMRI	Inconspicuous	B
	2^b^	Conventional MRI		
	3^a^	SyMRI		
	4^a^	Conventional MRI		

*HIE* hypoxic ischemic encephalopathy, *PVL* periventricular leukomalacia

^a^Novice rater

^b^Experienced rater

On both conventional and SyMRI-generated T1 and T2-weighted images, 29/32 (90.6%) cases were correctly diagnosed or identified as inconspicuous by raters 3 and 4 (Table 4). With respect to the assessment of less experienced raters, the sensitivity and specificity of conventional and SyMRI-generated MR contrasts differed (SyMRI: sensitivity: 0.81 [CI: 0.57–0.93]; specificity: 1 [CI 0.81–1]/conventional MRI: sensitivity: 0.94 [CI: 0.72–0.99]; specificity: 0.88 [CI: 0.64–0.96]; Fig. 4b).

The total values (all raters) for sensitivity and specificity differed between SyMRI-generated and conventionally acquired contrasts (SyMRI: sensitivity: 0.88 [CI: 0.72–0.95]; specificity: 1 [CI 0.89–1]/conventional MRI: sensitivity: 0.94 [CI: 0.80–0.98]; specificity: 0.94 [CI: 0.80–0.98]; Fig. 4c).

### Interrater Statistics

Based on the assessment of both conventionally acquired and SyMRI-generated T1-weighted and T2-weighted contrasts, there was a perfect agreement between the experienced raters, raters 1 and 2 (Cκ = 1 [CI: 0.99–1], *p* ≤ 0.001; [30]).

Based on the assessment of both conventionally acquired and SyMRI-generated T1-weighted and T2-weighted contrasts, there was a fair agreement between the less experienced raters, raters 3 and 4 (Cκ = 0.26 [CI: 0.25–0.27], *p* = 0.135; [30]).

On the basis of the assessment of both conventionally acquired and SyMRI-generated T1-weighted and T2-weighted contrasts, there was a moderate agreement between all raters (Fκ = 0.47 [CI: 0.32–0.61], *p* ≤ 0.001; [30]).

## Discussion

In this study the applicability of MR contrasts generated by the MR data post-processing software SyMRI was investigated in a clinical, radiological, diagnostic setting. Overall, SyMRI-generated T1-weighted and T2-weighted contrasts showed a lower sensitivity, but higher specificity, than conventional MR contrasts in a neonatal cohort (Fig. 4c). Interestingly, when sub-analyzing the evaluation by experienced raters, no differences between the two methods were found (Fig. 4a). In contrast, when evaluated by novice raters, conventionally acquired MR contrasts showed a higher sensitivity, whereas SyMRI-generated contrasts showed a higher specificity (Fig. 4b). Based on the data presented here, the application of SyMRI in neonates appears to be comparable to conventional T1-weighted and T2-weighted sequences and suitable in a clinical setting. In addition, further MR contrasts and quantitative image data are provided for radiological diagnostic assessment, without extending examination time and post-processing time.

As described in the literature, various types of brain damage that can occur in the neonatal period may severely affect further neurodevelopment [2, 31–34]. There is evidence that hemorrhage, hypoxic ischemic encephalopathy, and infarction are primarily among the most common causes of neonatal morbidity and mortality [35, 36]. These conditions are highly associated with preterm birth [37]. In order to best reflect the clinical reality, preterm infants and frequently observed pathologies were included in this study. In addition to conventionally acquired MR contrasts, SyMRI was applied to provide T1 and T2-weighted images. The software generates the contrasts based on the ascertained relaxation parameters and PD, determined by a single acquisition of an MDME sequence [16, 18].

These results are in line with several descriptions in the literature [15, 19]. The clinical application and the diagnostic accuracy of the method have already been investigated in neurological disorders in adults. It could be shown that contrasts generated by SyMRI were not inferior to those of conventionally acquired contrasts [15]; however, there are still few data on the use of this software in children and neonates. West et al. applied this technique in a cohort of 32 patients with a mean age of 12.6 years [38]. Furthermore, there are descriptions of the use of SyMRI in a cohort of 29 patients with a median age of 6 years [39]. Both studies showed that the method provides acceptable and reliable results [38, 39]. Nevertheless, on the basis of the assessment of novices, sensitivity and specificity differed and a poor agreement between both novice raters was observed. This finding is in accordance with descriptions in the literature [40, 41]. Since a perfect concordance for the evaluation of experienced neuroradiologists was observed, the poor agreement of novice raters was attributed to the lack of experience in the assessment of the neonatal brain [30]. Thus, based on the varying level of experience, there was a moderate agreement for the assessment of all raters [30].

Generally, experienced raters made more use of the possibility to modulate TR and TE, whereas novices tended to evaluate the images on the basis of the default preset values. Particularly when assessing myelination, an adaption of the scan parameters seems sensible. A short TR/TE MR contrast shows the presence of myelinated structures as hyperintense areas and enables the process of myelination in the course of development to be tracked. In contrast, a long TR/TE image contrast is appropriate for assessing the quantity of myelin deposited [42]. According to descriptions in the literature, TR/TE of 3000/120 ms is best suited for determining the individual state of brain maturity in the first months after birth. Using this adjustment, myelinated white matter appears considerably hypointense compared to non-myelinated brain areas and gray matter [42]. Hence, an adjustment of TR/TE facilitates the assessment of brain maturity. Furthermore, experienced raters preferred short TR/TE contrasts in case of suspected hypoxic ischemic encephalopathy, which is in line with descriptions by Barkovich et al. [43]. As shown in Table 4, novices misdiagnosed inconspicuous cases, as they suspected mild hypoxic ischemic encephalopathy. An adjustment of TR/TE might have enabled a more precise assessment. Based on the data presented here, no recommendations on the use of technical parameters for assessing the neonatal brain can be derived; however, SyMRI represents a method that enables the investigation of beneficial technical adjustments for the diagnosis of cerebral pathologies and the visualization of myelination in neonates.

Primarily in adults, the quality of SyMRI-generated images appears to be equal to that of conventional MRI sequences [15]. Also, in a pediatric cohort, there is evidence that the quality of the images was not inferior to conventionally acquired MR contrasts [38]. Interestingly, Andica et al. reported that fluid-attenuated inversion recovery (FLAIR) contrasts generated by SyMRI were even superior in the diagnosis of neonatal meningitis compared to conventional FLAIR sequences [44]. Our data show that MDME sequence-based MR post-processing allows diagnostic accuracy but in most cases the image quality was inferior to that of conventional MR images (Fig. 2). A possible explanation for this fact is that MR contrasts generated by SyMRI are based on a single MDME sequence. Hence, movement during the data acquisition presents as motion artifact in all MDME-based images; however, optimal conditions allow the generation of high-quality SyMRI image data in a neonatal cohort, as shown in Fig. 1.

This software offers advantages that may be of interest, especially when assessing the neonatal brain. Compared to conventional MR image data, quantitative MR mapping allows a better assessment of myelination and brain maturity [13, 17, 38]. Based on the relaxation constants of the examined tissue, voxels are assigned to gray and white matter, as well as to cerebrospinal fluid [13]; however, using conventional MR techniques, the generation of quantitative MR maps is a highly time-consuming process and, therefore, not applicable in the clinical routine. The software provides quantitative maps in a clinically acceptable time, which offers further prospects in medical imaging [17]; however, SyMRI-based quantitative MR mapping was beyond the scope of the present study and should be the subject of future investigations.

Nonetheless, an important weakness of this method must be pointed out. As shown in Table 4, based on conventional MR contrasts, the identification of subtle infarctions (Fig. 2c, g) is highly challenging even for experts in the field of neuropediatric radiology. The SyMRI does not provide DWI for the reliable diagnosis of stroke; however, further studies are needed to investigate whether SyMRI may provide information that proves beneficial for the detection of subtle cerebrovascular damage.

This study has several limitations. The technical properties of conventionally acquired and SyMRI-generated contrasts differed, especially regarding slice thickness and spatial resolution (Table 2). Hence, a direct comparison of both methods was limited to a certain extent. Due to the retrospective nature of the study, the sample size was relatively small. Furthermore, we included a high number of patients with hemorrhages, a condition that can be easily diagnosed, even by novices in the field of neonatal neuroradiology; however, it should be emphasized that hemorrhages represent one of the most common pathologies in preterm neonates [45].

In summary, our data indicate that SyMRI-generated T1-weighted and T2-weighted contrasts serve as a reliable tool for the detection of neonatal brain pathologies. The method demonstrated diagnostic non-inferiority compared to the current clinical gold standard. We conclude that the MR data post-processing software SyMRI is clinically applicable for the assessment of the neonatal brain, while leading to a more efficient use of available imaging time.

## Abbreviations

B1: Local radiofrequency field
CI: Confidence interval
Cκ: Cohen coefficient
DWI: Diffusion-weighted imaging
Fκ: Fleiss coefficient
FLAIR: Fluid-attenuated inversion recovery
MDME: Multi-dynamic multi-echo
MRI: Magnetic resonance imaging
PD: Proton density
SE: Spin echo
SWI: Susceptibility-weighted imaging
TE: Echo time
TR: Repetition time
TSE: Turbo spin echo
